# Immune Modulatory Effects of Human Chorionic Gonadotropin on Dendritic Cells Supporting Fetal Survival in Murine Pregnancy

**DOI:** 10.3389/fendo.2016.00146

**Published:** 2016-11-15

**Authors:** Dominique Dauven, Stefanie Ehrentraut, Stefanie Langwisch, Ana Claudia Zenclussen, Anne Schumacher

**Affiliations:** ^1^Department of Experimental Obstetrics and Gynecology, Medical Faculty, Otto-von-Guericke University, Magdeburg, Germany

**Keywords:** human chorionic gonadotropin, dendritic cells, regulatory T cells, fetal tolerance, pregnancy

## Abstract

Dendritic cells (DCs) are critically involved in the determination of immunity vs. tolerance. Hence, DCs are key regulators of immune responses either favoring or disfavoring fetal survival. Several factors were proposed to modulate DC phenotype and function during pregnancy. Here, we studied whether the pregnancy hormone human chorionic gonadotropin (hCG) is involved in DC regulation. *In vitro*, bone marrow-derived DCs (BMDCs) were stimulated in the presence or absence of urine-purified or recombinant hCG (rhCG) preparations. Subsequently, BMDC maturation was assessed. Cytokine secretion of activated BMDCs and their capability to enforce TH1, TH2, TH17, or Treg cell differentiation was determined after rhCG treatment. Moreover, the *in vivo* potential of hCG-modulated BMDCs to influence pregnancy outcome, Treg cell number, and local cytokine expression was evaluated after adoptive transfer in a murine abortion-prone model before and after conception. Both hCG preparations impaired the maturation process of BMDCs. rhCG treatment did neither alter cytokine secretion by BMDCs nor their ability to drive TH1, TH2, or TH17 differentiation. rhCG-treated BMDCs augmented the number of Treg cells within the T cell population. Adoptive transfer of rhCG-treated BMDCs after conception did not influence pregnancy outcome. However, transfer of hCG-treated BMDCs prior to mating had a protective effect on pregnancy. This positive effect was accompanied by increased Treg cell numbers and decidual IL-10 and TGF-β expression. Our results unveil the importance of hCG in retaining DCs in a tolerogenic state, thereby promoting Treg cell increment and supporting fetal survival.

## Introduction

Dendritic cells (DCs) are situated at the interface between the innate and the adaptive immune system and are, therefore, critically involved in the initiation or suppression of adaptive immune responses. During pregnancy, the immune system faces a dilemma. On the one hand, it has to grant immunity against potential harmful pathogens while, at the same time, it has to tolerate the foreign antigens expressed by the semi-allogeneic fetus. As DCs are key regulators in the balance between tolerance and immunity, they are proposed to be pivotal players in the decision whether the fetus is tolerated or rejected.

Human DCs represent approximately 1.7% of all CD45^+^ cells in the decidua and the vast majority displays a myeloid phenotype (1). In normal pregnancy, Kämmerer and colleagues described a unique DC population in the human decidua displaying an immature phenotype with a high proliferative potential and low T cell stimulatory capacity (2). Another study identified a subset of tolerogenic DCs in human decidual tissue, which produce high amounts of IL-10 (3). In mice, an accumulation of DCs has been reported in the receptive phase of the estrus cycle in uterine tissue even before pregnancy arises. DC clusters were located close to the uterine lumen or small blood vessels on the anti-mesometrial side (4). During normal murine pregnancy, the highest number of total DCs could be detected at early pregnancy stages where the average density of CD11c^+^ cells was 1.3% of all nucleated cells in the decidua. Within the total DC pool, the relative percentage of lymphoid DCs was highest at mid gestation, while the relative percentage of myeloid DCs was highest at early and late gestation (5). One study by Plaks and colleagues confirmed the indispensable role of murine uterine DCs for the implantation process (6), while two other human studies proposed an association between an elevated number of mature DCs and recurrent spontaneous abortions (7, 8). Thus, it is tempting to speculate that dysregulations in the number of different DC subpopulations and their maturation states may positively or negatively influence pregnancy outcome. However, the factors and underlying mechanisms affecting the phenotype and function of DCs during pregnancy are still not completely understood.

Several human and murine studies proposed trophoblast-associated factors as potential candidates in DC modulation (9, 10). This includes factors, such as soluble thymic stromal lymphopoitin and pregnancy-specific glycoprotein (11, 12). After being in contact with trophoblasts, DCs have been shown to retain an immature phenotype, produce IL-10 and TGF-β rather than IL-12 and TNF-α, have a reduced capability to stimulate allogeneic immune responses, and enforce TH2 differentiation and Treg cell induction (9, 11, 12). These mechanisms are all involved in fetal tolerance.

Besides these, female sex hormones such as progesterone and estrogen were implied to impact DCs. Hormonal stimulation of activated bone marrow-derived DCs (BMDCs) resulted in the majority of studies in an impaired upregulation of MHCII molecules and costimulatory molecules associated with a decreased potential to secrete pro-inflammatory cytokines. In line with a hormonal-driven induction of a tolerogenic phenotype in activated BMDCs, their T cell stimulatory capacity was reduced (13, 14). However, these data are contrary to findings obtained after hormonal stimulation of human monocyte-derived DCs (15, 16). These studies reported rather an upregulation of IL-10 in hormone-treated DCs and a TH2-promoting capacity than an effect on maturation markers. We have recently investigated the influence of two other pregnancy hormones, namely human chorionic gonadotropin (hCG) and luteinizing hormone (LH) on the number and phenotype of peripheral and local DCs in a murine model of disturbed fetal tolerance. The application of both hormones *in vivo* prevented fetal rejection and was associated with a reduced number of total and mature DCs in the decidua. Moreover, there is evidence that decidual DCs isolated from hCG-treated animals are able to induce Treg cell generation (17). Consistently with our observation, other studies described an effect of hCG on human and murine DCs (15, 18–21). However, the *in vivo* contribution of hCG-modulated DCs remains mainly unclear.

In our current study, we aimed to understand the impact of hCG, another trophoblast-secreted factor, on DCs. By performing several *in vitro* experiments, we first analyzed the potential of two hCG preparations, urine-purified hCG (uhCG) and recombinant hCG (rhCG), to affect maturation, cytokine secretion, and T cell differentiation capability of BMDCs. We decided to additionally include rhCG in our study, as this hCG preparation displays some fundamental advantages compared to uhCG, and we were interested to assess potential differences between both preparations. Afterward, we studied the influence of hCG-modulated BMDCs on pregnancy outcome in an *in vivo* setting.

## Materials and Methods

### Animals

CBA/J females and DBA/2J males were purchased from Charles River (France) and Janvier Labs (France). All animals were maintained in our animal facility and treated according to the institutional guidelines with the ministerial approval (Landesverwaltungsamt Sachsen-Anhalt AZ42502-2-1125 UNIMD). The experiments were conducted by authorized persons according to the Guide for Care and Use of Animals in Agriculture Research and Teaching. Mice were kept under a 12-h light/12-h dark cycle, and water and food was provided *ad libitum*. Virgin CBA/J females at the age of 8 weeks were used for bone marrow (BM) isolation or mated with 8- to10-week old DBA/2J males. DBA/2J-mated CBA/J females are known to spontaneously develop high abortion rates and are, therefore, used as abortion-prone females. After mating, females were checked for vaginal plug twice a day, whose appearance indicated day 0 of pregnancy. On day 12 of pregnancy, females were sacrificed for determination of the abortion rate, number of implantations, and for tissue processing.

### Generation of BMDCs

Bone marrow-derived DCs were generated using a common protocol described in the literature (22) with modifications. Briefly, femora and tibiae were removed from virgin CBA/J females and flushed with 2 ml Dendritic Cell Medium [DCM; RPMI 1640 (Invitrogen, Germany) supplemented with 10% fetal bovine serum (Biochrom, Germany), 1% penicillin/streptomycin (Invitrogen, Germany), and 50 μM 2-mercaptoethanol (Sigma, Germany)]. The cell suspension was centrifuged and erythrocytes were lysed before filtering the cells through a 100 μm sieve. Cells were suspended in 20 ml DCM enriched with 10 ng/ml granulocyte macrophage colony-stimulating factor (GM-CSF; PeproTech, Germany). Non-adherent cells were harvested after 2 h of incubation and were further cultured at a concentration of 3 × 10^6^ cells per well in GM-CSF-enriched DCM at 37°C and 5% CO_2_. On day 3, 40% of the medium was refreshed. Two days later non-adherent cells were harvested and re-suspended in fresh GM-CSF-enriched DCM. On day 7, BMDCs were collected for *in vitro* stimulation.

### *In Vitro* Stimulation of BMDCs

The 8 × 10^5^ BMDCs were cultured in 500 μl DCM per well. To induce maturation, BMDCs were stimulated with 2 μg/ml lipopoly-saccharide (LPS; Sigma, Germany) and 200 ng/ml interferon-γ (IFN-γ; Invitrogen, Germany). To study the effect of hCG on DC maturation, BMDCs were additionally treated with various concentrations of either uhCG (Pregnyl; EurimPharm, Germany; 100, 250, and 500 IU/ml) or rhCG (Ovitrelle, MerckSerono, UK; 50, 100, and 500 mIU/ml). BMDCs cultured in DCM only served as controls. After 24 h of stimulation, BMDCs and cell culture supernatants were collected for flow cytometry or cytometric bead array (CBA) analysis.

### Cocultures of BMDCs and T Cells

The 8 × 10^5^ BMDCs in 500 μl DCM were stimulated with LPS and IFN-γ in the presence or absence of 500 mIU/ml rhCG for 24 h. Unstimulated BMDCs cultured in DCM only served as controls. Naïve CD4^+^CD25^−^ T cells were isolated from virgin CBA/J females using the Regulatory T cell Isolation Kit (Miltenyi Biotec, Germany), following the instructions provided by the manufacturer. To study the potential of the treated BMDCs to drive T cell differentiation, 2 × 10^5^ BMDCs and 2 × 10^5^ T cells were cocultured in DCM containing 10 ng/ml IL-2, 1 μg/ml anti-CD3, and 5 μg/ml anti-CD28 (R&D Systems, Germany) for 48 h. Five hours before cell harvest, 50 ng/ml phorbol 12-myristate 13-acetate (Sigma, Germany), 500 ng/ml Ionomycin (Sigma, Germany), and 10 μg/ml Brefeldin A (Biolegend, UK) were added to the cocultures. Afterward, the number of TH1, TH2, TH17, and Treg cells was determined by flow cytometry.

### Adoptive Transfer of BMDCs

Bone marrow-derived DCs were stimulated *in vitro* and then adoptively transferred into CBA/J females. More precisely, 1 × 10^7^ BMDCs were stimulated with LPS and IFN-γ in the presence or absence of 500 mIU/ml rhCG for 24 h. Afterward, 1 × 10^6^ BMDCs in PBS (PAA Laboratories, Germany) were injected intravenously. In the first set of experiments, CBA/J females previously mated to DBA/2J males were injected on gestational day 1–3. In the second set of experiments, virgin CBA/J females were adoptively transferred with the cells and immediately mated to DBA/2J males. CBA/J females that did not become pregnant within 3 days were excluded from the experiment.

### Tissue Sampling and Isolation of Mononuclear Cells

On day 12 of pregnancy, blood was obtained from CBA/J females by retroorbital puncture under anesthesia. Afterward, females were sacrificed by cervical dislocation. Spleen, thymus, and para-aortic lymph nodes were removed, washed in ice cold PBS, and kept in RPMI 1640 medium at 4°C. Pregnant uteri were opened longitudinally and the number of implantations as well as the abortion rates were documented. Fetoplacental units were separated from their implantation sites, deciduas were cut in small pieces and collected in HBSS without Ca^2+^ and Mg^2+^ (Sigma, Germany). Additionally, one piece of decidua was snap frozen for Real-time RT-PCR analysis. Mononuclear cells from blood, spleen, thymus, para-aortic lymph nodes, and decidua samples were isolated using our established protocol (23) and analyzed by flow cytometry.

### Flow Cytometry Analysis

Bone marrow-derived DCs and T cells from *in vitro* assays as well as mononuclear cells obtained from pregnant females were stained for extra- and intracellular markers as described elsewhere (23). The following antibodies were used: FITC-conjugated anti-mouse CD80 (clone 16-10A1), PE-conjugated anti-mouse I-A/I-E (clone: M5/114.15.2), FITC-conjugated anti-mouse CD4 (clone: RM4-4), PE-conjugated anti-mouse Foxp3 (clone: FJK-16s), PE-conjugated anti-mouse TNFα (clone: MP6-XT22), PerCP-conjugated anti-mouse IFN-γ (clone: XMG1.2), PE-conjugated anti-mouse IL-17 (clone: TC11-18H10), PE-conjugated anti-mouse IL-4 (clone: 11B11), and APC-conjugated anti-mouse IL-10 (clone: JES5-16E3). Foxp3 antibody was purchased from eBioscience, Germany. All other antibodies were purchased from BD Biosciences, Germany.

### Cytokine Determination by CBArray Analysis

To quantify cytokines in supernatants of BMDC cultures, the CBArray kit for IL-6, IL-10, MCP-1, TNF, and IL-12p70 (BD Biosciences, Germany) was used according to the instructions provided by the manufacturer. Briefly, cytokine capture beads were mixed with 50 μl of supernatants and incubated for 2 h with PE-conjugated detection antibodies to form sandwich complexes. After washing, the complexes were re-suspended and measured by flow cytometry.

### Real-Time RT-PCR

Frozen decidual tissue was treated with Trizol^®^ Reagent (Invitrogen, Germany) and disaggregated using a homogenizer (Ultra-Turrax T8; IKA, Germany). Isolation of RNA, cDNA synthesis, and real-time RT-PCR were performed as described elsewhere (24). For detection of IL-10 and TGF-β TaqMan technology was conducted using an iQ5 Multicolor RT-PCR Detection System (Bio-Rad Laboratories, Germany). β-Actin was employed as housekeeping gene and relative gene expression was calculated by 2^−ΔCT^. Primer and probe sequences are available upon request.

### Data Analysis and Statistics

Analysis of data was performed using the GraphPad Prism 5.0 software (STATCON, Germany). Data obtained from *in vitro* assays are presented as means plus SEM. *In vivo* data and RT-PCR data are presented as medians in graphs showing individual values for each animal. *In vitro* data were analyzed using one-way ANOVA followed by Bonferroni correction for multiple comparisons. All other data were analyzed using the non-parametric Mann–Whitney *U* test. In all cases, *p* < 0.05 was considered to be statistical significant.

## Results

### *In Vitro* Maturation of BMDCs Was Hampered by rhCG and uhCG

**Figure 1:**
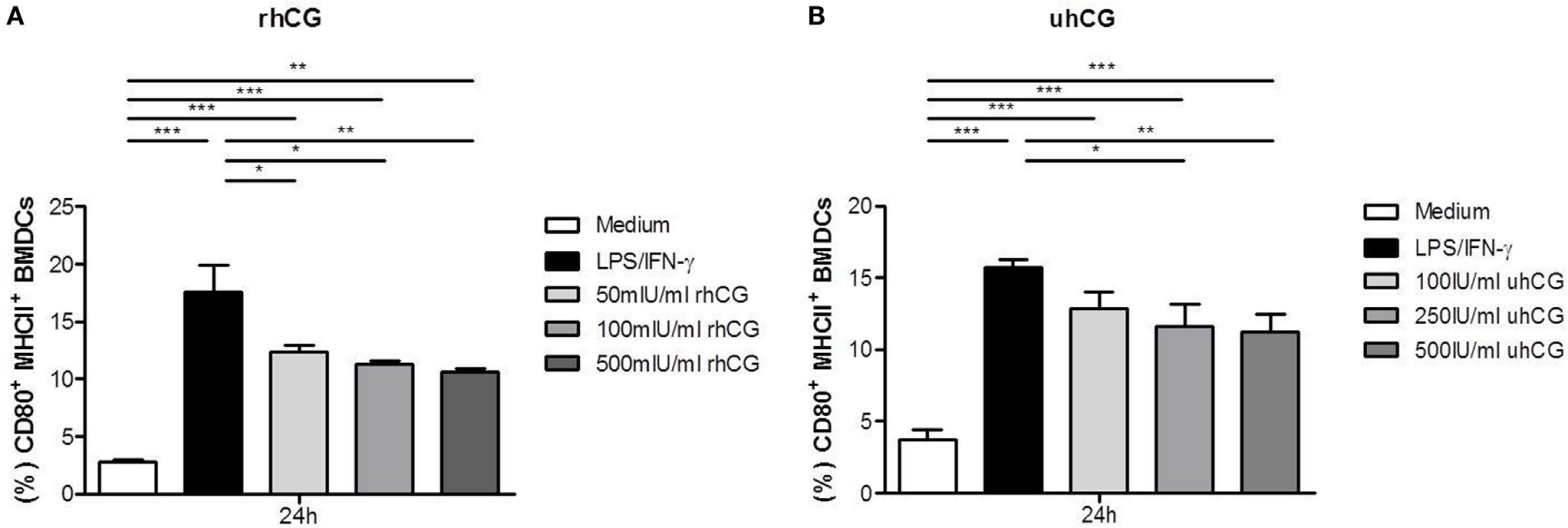
**Treatment with rhCG and uhCG impaired BMDC maturation**. BMDCs were stimulated with LPS/IFN-γ in the presence or absence of different concentrations of rhCG **(A)** or uhCG **(B)** for 24 h. Unstimulated BMDCs served as controls. BMDC maturation was assessed by MHCII and CD80 expression *via* flow cytometry. Both hCG preparations hampered the maturation of activated BMDCs in a concentration-dependent manner. Each assay was repeated five times in duplicates. Data are presented as means plus SEM. Statistical analysis was carried out by one-way ANOVA followed by Bonferroni correction for multiple comparisons. *p < 0.05, **p < 0.01, ***p < 0.001.

Previously, Wan and colleagues showed that uhCG interferes with BMDC maturation. Here, in addition to uhCG, we tested the effects of rhCG on BMDCs stimulated with LPS and IFN-γ for 24 h. Both hCG preparations were used to study a concentration-dependent effect. After 24 h of culture, we observed a significant increase in the number of mature CD80^+^MHCII^+^ BMDCs after LPS and IFN-γ stimulation (Figures 1A,B). In the presence of 50, 100, and 500 mIU/ml rhCG, the number of mature BMDCs expressing CD80 and MHCII was significantly reduced when compared to BMDCs that were not treated with hCG (Figure 1A). In line, 250 and 500 IU/ml uhCG significantly hampered BMDC maturation (Figure 1B). A lower concentration of uhCG (100 IU/ml), decreased, although not significantly, CD80^+^MHCII^+^ BMDC numbers.

### Treatment with rhCG Did Not Alter Cytokine Secretion of BMDCs

**Figure 2:**
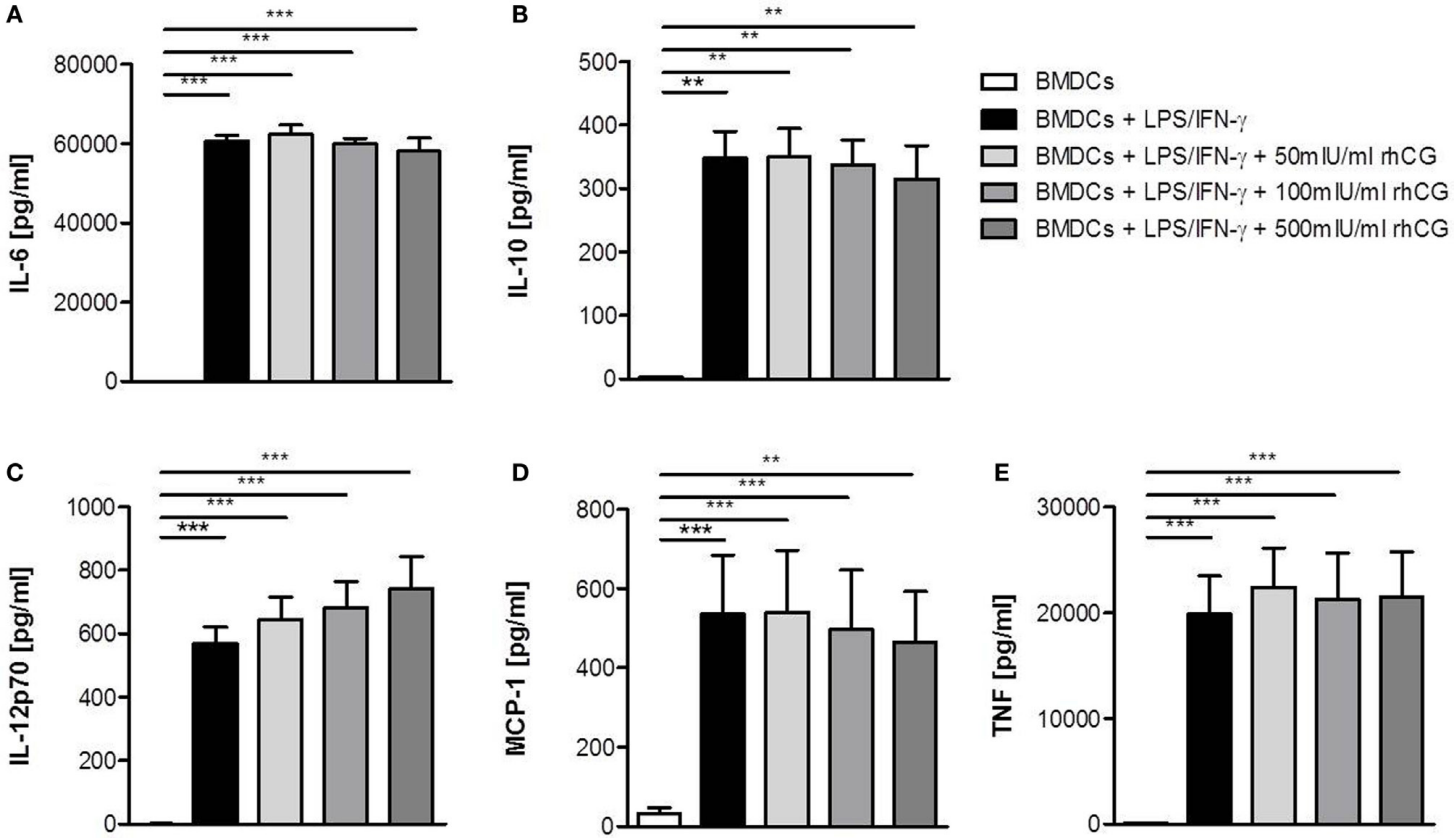
**Treatment with rhCG did not alter cytokine secretion by activated BMDCs**. BMDCs were stimulated with LPS/IFN-γ in the presence or absence of different concentrations of rhCG for 24 h. Unstimulated BMDCs served as controls. Secretion of IL-6 **(A)**, IL-10 **(B)**, IL-12p70 **(C)**, MCP-1 **(D)**, and TNF **(E)** was determined by CBArray analysis *via* flow cytometry. rhCG treatment did not influence the cytokine secretion profile of BMDCs. Each assay was repeated five times in duplicates. Data are presented as means plus SEM. Statistical analysis was carried out by one-way ANOVA followed by Bonferroni correction for multiple comparisons. **p < 0.01, ***p < 0.001.

After we confirmed that rhCG had the same potential to interfere with BMDC maturation as uhCG, we focused our study on rhCG and evaluated whether the addition of hCG to the culture alters cytokine secretion by LPS/IFN-γ-stimulated BMDCs. BMDCs were, therefore, cultured in the presence or absence of different concentrations of rhCG for 24 h and analyzed for their secretion of IL-6, IL-10, IL-12p70, MCP-1, and TNF. None of the rhCG concentrations did significantly influence the secretion of any of the investigated cytokines (Figures 2A–E).

### rhCG-Treated BMDCs Did Not Influence the Differentiation of Naïve T Cells in TH1, TH2, and TH17 Cells

**Figure 3:**
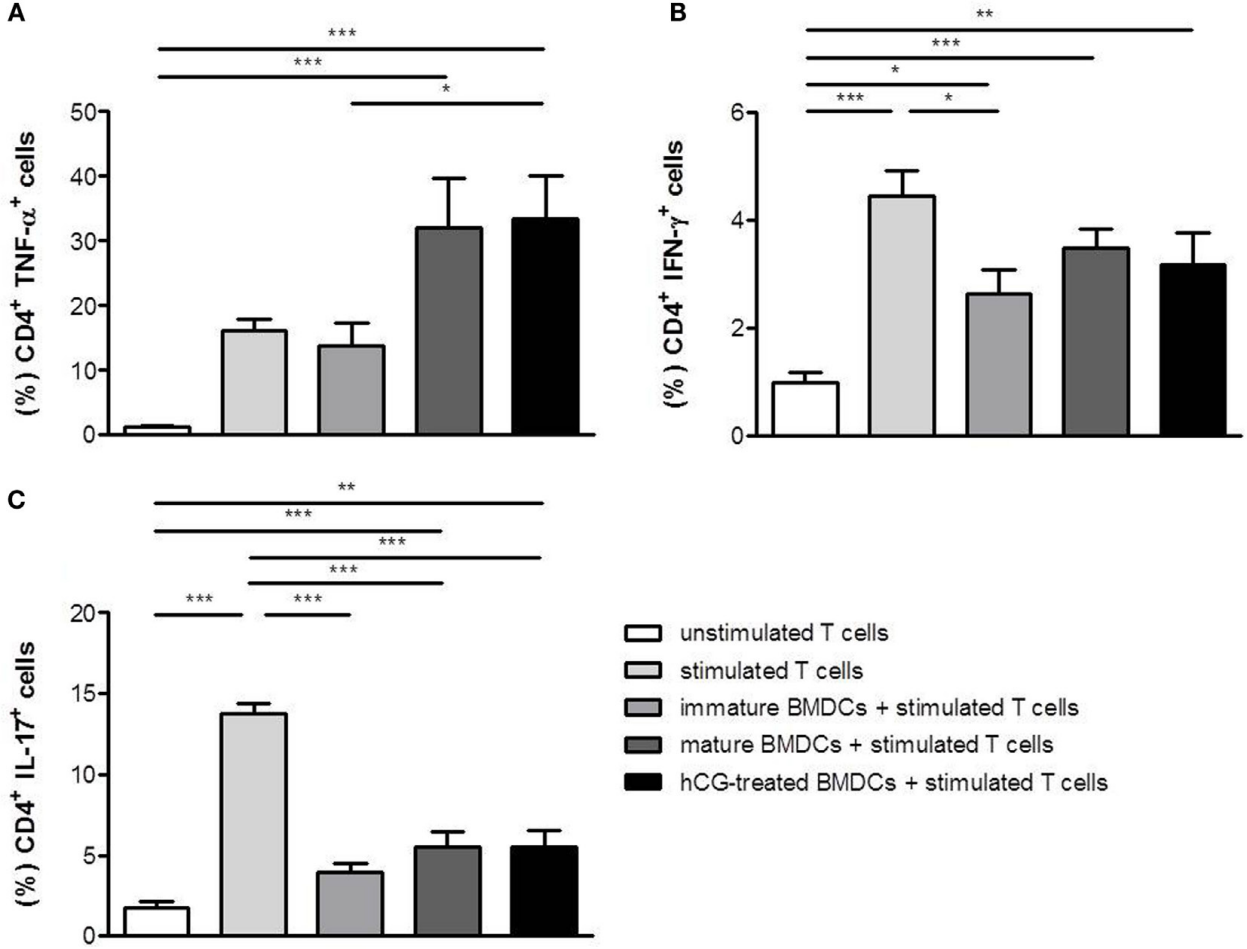
**Treatment with rhCG did not affect TH1 and TH17 differentiation capacity of BMDCs**. BMDCs were stimulated with LPS/IFN-γ in the presence or absence of 500 mIU/ml rhCG for 24 h or left unstimulated (immature BMDCs). Afterward, BMDCs were cocultured with stimulated naïve T cells for 48 h. Unstimulated T cells served as controls. T cell differentiation was evaluated by the expression of the type-1 cytokines TNF-α **(A)** and IFN-γ **(B)** or the type-17 cytokine IL-17 **(C)** *via* flow cytometry. Treatment with rhCG did not influence TH1 and TH17 differentiation. Each assay was repeated six times in duplicates. Data are presented as means plus SEM. Statistical analysis was carried out by one-way ANOVA followed by Bonferroni correction for multiple comparisons. *p < 0.05, **p < 0.01, ***p < 0.001.

Next, we were interested to analyze whether BMDCs previously treated with rhCG were able to influence T cell differentiation *in vitro*. BMDCs were stimulated with LPS/IFN-γ, cultured in the presence or absence of rhCG or left unstimulated (immature BMDCs). Afterward, BMDCs were cocultured with naïve T cells for 48 h to assess their ability to drive T cell differentiation toward type-1, type 2, or type-17. In the absence of BMDCs, T cell activation *via* the TCR resulted in a strong upregulation of the TH1 cytokines TNF-α and IFN-γ as well as IL-17 (Figures 3A–C). IFN-γ and IL-17 upregulation was impaired in the presence of immature BMDCs (Figures 3B,C). Notably, mature BMDCs without hCG treatment further increased TNF-α upregulation in T cells (Figure 3A). hCG-treated BMDCs did not significantly influence the increase in TNF-α, IFN-γ, and IL-17 expression when compared to mature BMDCs without hCG treatment (Figures 3A–C).

**Figure 4:**
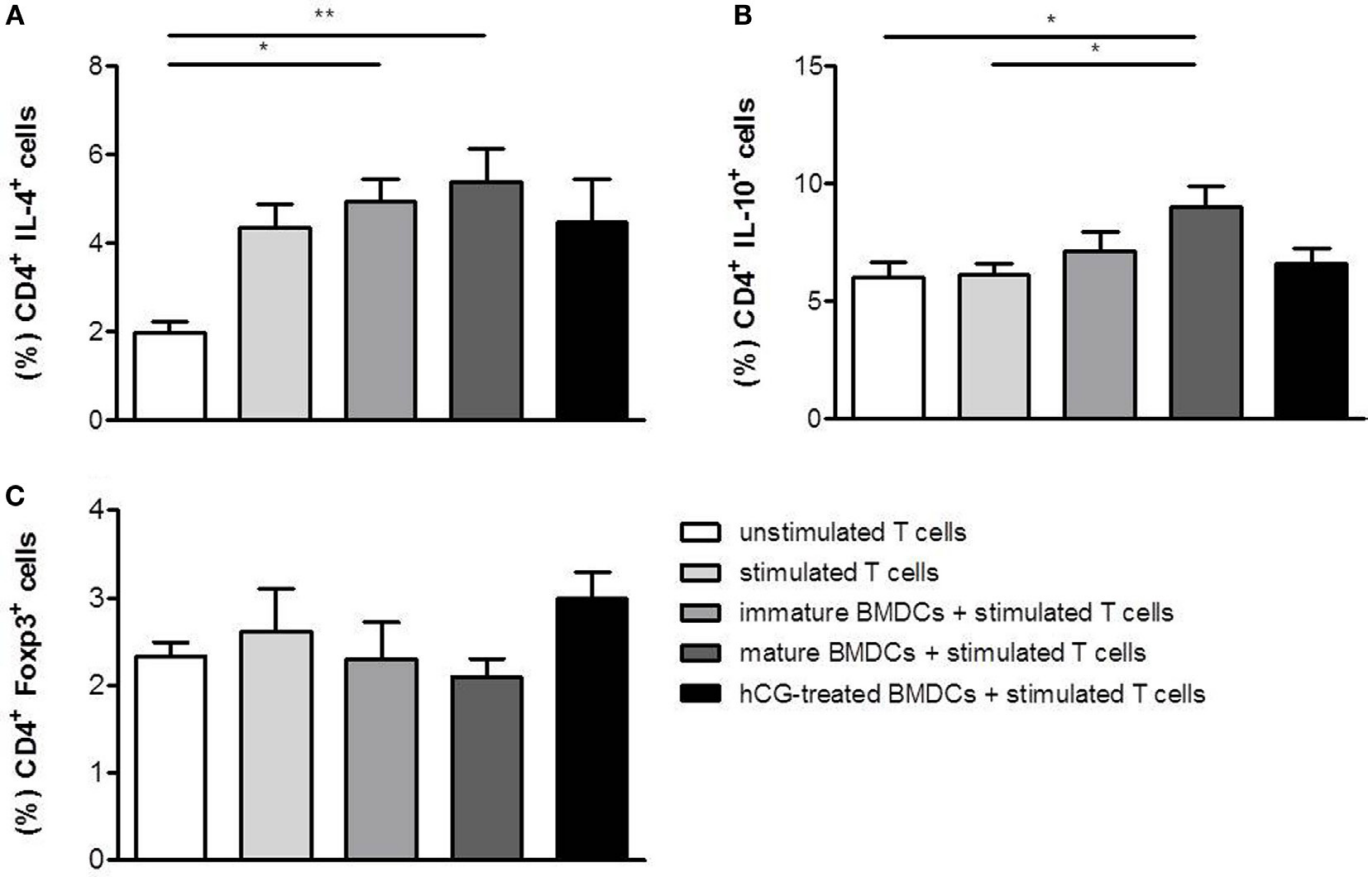
**Treatment with rhCG did not alter TH2 differentiation capacity of BMDCs had a modest effect on the potential of BMDCs to induce Treg cells**. BMDCs were stimulated with LPS/IFN-γ in the presence or absence of 500 mIU/ml rhCG for 24 h or left unstimulated (immature BMDCs). Afterward, BMDCs were cocultured with stimulated naïve T cells for 48 h. Unstimulated T cells served as controls. T cell differentiation was evaluated by the expression of the type-2 cytokines IL-4 **(A)** and IL-10 **(B)**, and the number of CD4^+^ Foxp3^+^ Treg cells **(C)** was determined *via* flow cytometry. Treatment with rhCG did not alter TH2 polarization by BMDCs but induced a modest elevation of Treg cells. Each assay was repeated six times in duplicates. Data are presented as means plus SEM. Statistical analysis was carried out by one-way ANOVA followed by Bonferroni correction for multiple comparisons. *p < 0.05, **p < 0.01.

As for TH2 cytokines, T cell activation resulted in an upregulation of IL-4, but not IL-10. The presence of both, immature and mature BMDCs, further increased IL-4 and IL-10 expression in stimulated T cells (Figures 4A,B). hCG-treated BMDCs did not provoke any statistically significant changes in secretion of IL-4 and IL-10 in T cells when compared to mature BMDCs (Figures 4A,B). Hence, no changes in T cell differentiation were observed when naïve T cells were cocultured with hCG-treated BMDCs.

### rhCG-Treated BMDCs Provoked a Modest Augmentation of CD4^+^Foxp3^+^ Treg Cells within the Naïve T Cell Population

In addition to a potential impact on T cell differentiation, we evaluated whether BMDCs may influence the generation of Treg cells after they have been treated with rhCG. Therefore, BMDCs were stimulated for 24 h with LPS/IFN-γ, cultured in the absence or presence of rhCG or left unstimulated (immature BMDCs). Afterward, BMDCs were cocultured with naïve T cells. BMDCs did not significantly influence Foxp3 expression in naïve T cells (Figure 4C). However, rhCG-treated BMDCs induced a modest elevation of CD4^+^ T cells expressing Foxp3 when compared to mature BMDCs without hCG treatment (Figure 4C).

### Adoptive Transfer of rhCG-Treated BMDCs into Abortion-Prone Females after Conception Did Not Affect Fetal Resorption

**Figure 5:**
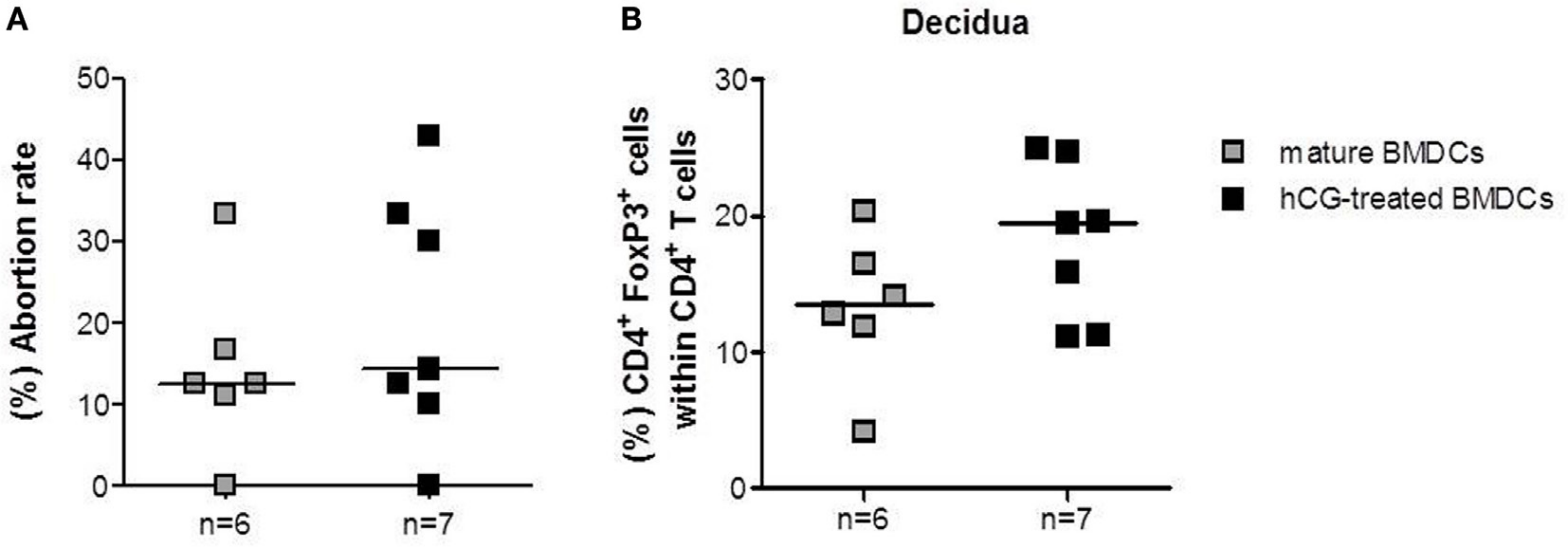
**Adoptive transfer of rhCG-treated BMDCs into abortion-prone females after conception did not affect pregnancy outcome**. BMDCs were stimulated with LPS/IFN-γ in the presence or absence of 500 mIU/ml rhCG for 24 h. Afterward, 1 × 10^6^ BMDCs were adoptively transferred into pregnant abortion-prone females at days 1–3 of gestation. On day 12 of pregnancy, females were sacrificed and the pregnancy outcome **(A)** as well as the number of decidual Treg cells **(B)** was determined *via* flow cytometry. Transfer of rhCG-treated BMDCs elevated the number of decidual Treg cells but did not influence the abortion rate. Data are presented as medians. Individual values are displayed for each animal. Statistical analysis was carried out by using the non-parametric Mann–Whitney *U* test.

After we provided evidence that rhCG hampered BMDC maturation, we aimed to study the *in vivo* effects of hCG-treated BMDCs in a murine model of disturbed fetal tolerance. Based on the current literature, we hypothesized that adoptive transfer of hCG-induced tolerogenic BMDCs into abortion-prone females may result in an *in vivo* elevation of Treg cells and thereby prevent fetal rejection. According to our previous protocol for a transfer of Treg cells (23), we adoptively transferred BMDCs during early pregnancy stages (gd 1–3). On day 12 of pregnancy, we found an augmented number of CD4^+^ Foxp3^+^ Treg cells in the decidua of abortion-prone females that received hCG-treated BMDCs when compared to females that received mature BMDCs (Figure 5B). However, changes in the number of Treg cells were not associated with positive effects in pregnancy outcome as the abortion rate remained unchanged (Figure 5A). The number of implantations was also comparable between both groups (data not shown). In thymus (median: 0.63 vs. 0.92), spleen (median: 11.40 vs. 12.69), para-aortic lymph nodes (median: 8.09 vs. 8.21), and blood (median: 4.45 vs. 4.99) we detected comparable Treg cell numbers between females that received mature BMDCs or hCG-treated BMDC, respectively.

### Adoptive Transfer of hCG-Treated BMDCs into Abortion-Prone Females Prior to Mating Significantly Increased Treg Cell Number and Diminished Fetal Rejection

**Figure 6:**
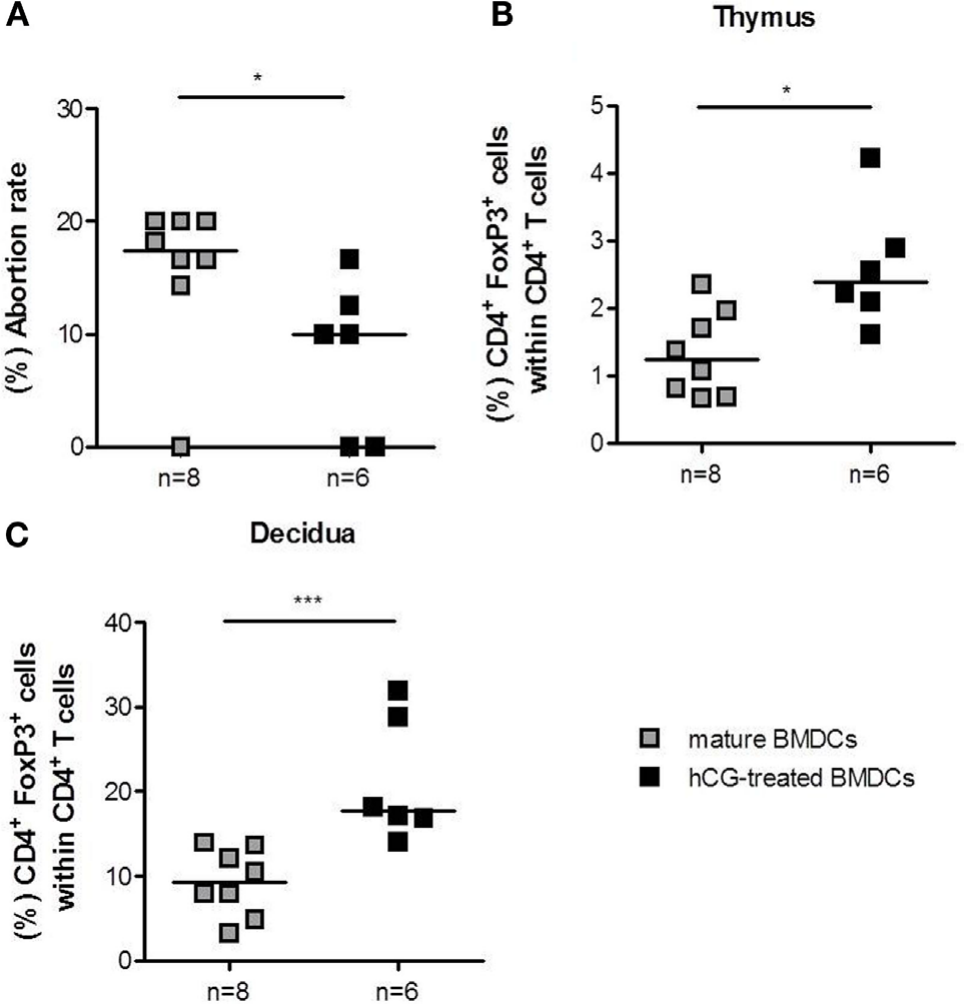
**Adoptive transfer of hCG-treated BMDCs into abortion-prone prior to mating diminished fetal resorption**. BMDCs were stimulated with LPS/IFN-γ in the presence or absence of 500 mIU/ml rhCG for 24 h. Afterward, 1 × 10^6^ BMDCs were adoptively transferred into virgin animals immediately before mating. On day 12 of pregnancy, females were sacrificed, and the pregnancy outcome **(A)** as well as the number of thymic **(B)** and decidual **(C)** Treg cells was determined *via* flow cytometry. Transfer of hCG-treated BMDCs significantly increased the number of thymic and decidual Treg cells. This was associated with a diminution of fetal resorption in the hCG-treated BMDC group. Data are presented as medians. Individual values are displayed for each animal. Statistical analysis was carried out by using the non-parametric Mann–Whitney *U* test. *p < 0.05, ***p < 0.001.

As the transfer of hCG-treated BMDCs after conception did not significantly influence Treg cell number and pregnancy outcome, we hypothesized that the time point for adoptive transfer was too late. Previous results indicated that the depletion of Treg cells prior to mating drastically impairs the implantation process and lead to an infiltration of activated T effector cells (25). Furthermore, adoptive transfer of Treg cells in abortion-prone animals is only protective when performed between gestation days 0–2 (23), while Treg cell depletion at later pregnancy stages only provokes a minor increase in fetal resorption (26). These data underline the need for Treg cells at very early pregnancy stages to guarantee proper implantation and fetal development. Hence, we decided to transfer BMDCs prior to mating to achieve early Treg cell elevation. CD4^+^Foxp3^+^ Treg cell numbers in spleen (median: 10.88 vs. 10.43), para-aortic lymph nodes (median: 8.96 vs. 11.15), and blood (median: 5.01 vs. 4.83) did not differ statistically between abortion-prone females that received mature BMDCs or hCG-treated BMDCs, respectively. The application of hCG-treated BMDCs, however, did significantly elevate thymic and decidual Treg cell frequency (Figures 6B,C). Notably, application of hCG-treated BMDCs resulted in a significant reduction in the abortion rate compared to mature BMDCs (Figure 6A), while both groups showed comparable numbers of implantations (data not shown).

### Protective Effect of rhCG-Treated BMDCs Was Associated with Elevated TGF-β and IL-10 Levels at the Fetal–Maternal Interface

**Figure 7:**
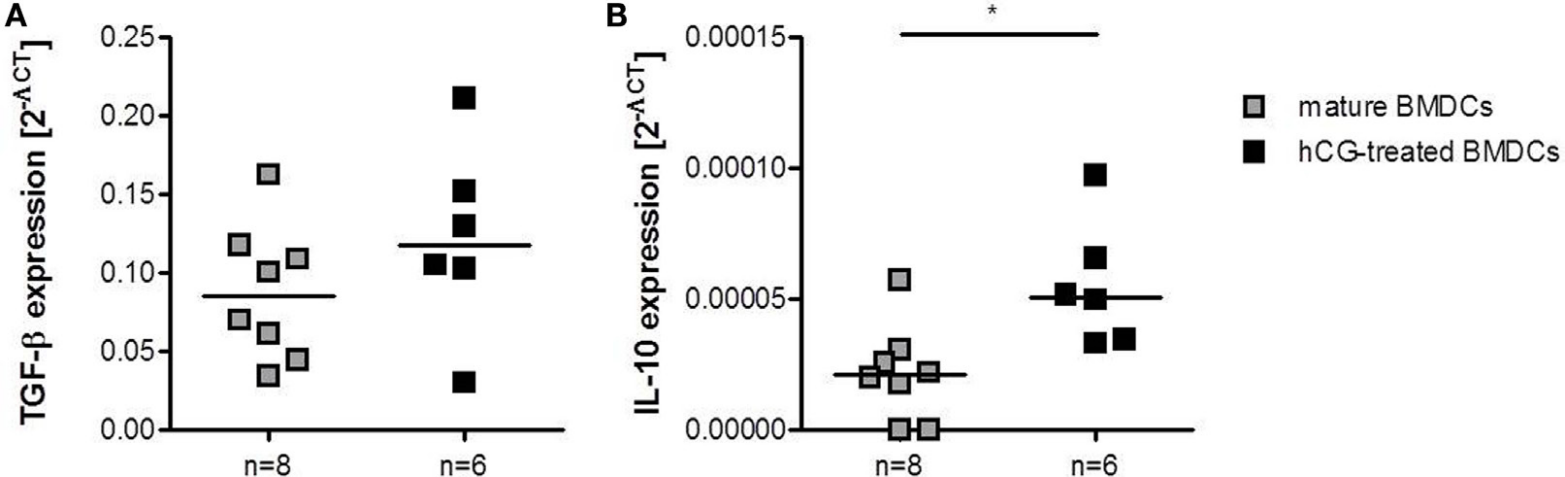
**Adoptive transfer of hCG-treated BMDCs into abortion-prone females prior to mating augmented decidual IL-10 and TGF-β expression**. BMDCs were stimulated with LPS/IFN-γ in the presence or absence of 500 mIU/ml rhCG for 24 h. Afterward, 1 × 10^6^ BMDCs were adoptively transferred into virgin animals immediately before mating. On day 12 of pregnancy, females were sacrificed and decidual expression of TGF-β and IL-10 was assessed by RT-PCR. Transfer of hCG-treated BMDCs increased the expression of TGF-β **(A)** and IL-10 **(B)** when compared to females treated with mature BMDCs. Data are presented as medians. Individual values are displayed for each animal. Statistical analysis was carried out by using the non-parametric Mann–Whitney *U* test. *p < 0.05.

Finally, we analyzed whether adoptive transfer of hCG-treated BMDCs affected the local mRNA expression of TGF-β and IL-10, two anti-inflammatory molecules that have been associated with Treg cell function (27). In accordance to the augmented number of Treg cells, we found elevated TGF-β and IL-10 levels in decidual tissue of abortion-prone females that received hCG-treated BMDCs as compared to females that received mature BMDCs (Figures 7A,B).

## Discussion

Dendritic cells are critically involved in fetal fate as they are key regulators of pro- and anti-fetal immune responses. These cells are implicated in important immune regulatory processes at different pregnancy stages.

Trophoblast-associated factors have been proposed to induce tolerogenic DCs. Within these factors, hormones, such as progesterone and estrogen, seem to play a major role in DC regulation. Interestingly, DCs derived from various origins, such as BM, monocytes, spleen, or decidua, are affected by hormonal stimulation [summarized in Ref. (28)]. Recently, we suggested a role for hCG in modulating DC phenotype and function in murine pregnancy after observing that *in vivo* hCG application to abortion-prone females retained uterine DCs in a rather immature state, further promoting Treg cell induction and preventing immunological rejection of the fetus (17). However, the precise mechanisms occurring *in vivo* after hCG application remained unclear. Thus, our present study intended to shed more light on the direct effects of hCG on DCs. In the first set of *in vitro* experiments, we analyzed whether two hCG preparations, uhCG and rhCG, are able to influence the maturation process, cytokine secretion, and T cell differentiation capability of BMDCs. In a previous publication, Wan and colleagues reported that the treatment of LPS/IFN-γ-activated BMDCs with uhCG blocked MHCII upregulation, but did not alter the expression of CD80 and CD11c (20). Using an uhCG preparation from the same supplier we observed similar results. Additionally, we decided to test the efficacy of rhCG in our study as the usage of recombinant preparations display some advantages compared to urine preparations. This is primarily due to potential contaminations by non-hCG proteins in urine preparations that may influence the results. Second, uhCG preparations have high batch-to-batch variations that may decrease the reproducibility of the experiments. Moreover, in a recent study, we found the rhCG preparation to be slightly more potent in inducing Treg cells than the urine preparation at least *in vitro* (29). Here, we observed a significantly impaired upregulation of MHCII and CD80 expression on LPS/IFN-γ-activated BMDCs in the presence of rhCG suggesting that rhCG is as effective as uhCG in blocking BMDC maturation. Based on these results and on the aforementioned experimental disadvantages of uhCG, we decided to use rhCG in all the following experiments. The blocking effect on BMDC maturation became stronger with increasing concentrations of both hCG preparations suggesting a concentration-dependent impact of hCG on BMDCs.

Secretion of different pro-inflammatory cytokines as well as the production of the anti-inflammatory cytokine IL-10 was not altered in the presence of any rhCG concentration tested. This contradicts the study by Wan and colleagues, who observed a significant elevation of IL-6, IL-10, and IL-12 after uhCG treatment of activated BMDCs (20). In our study, we did not analyze cytokine secretion of BMDCs after treatment with uhCG. However, we may speculate that cytokine secretion by activated BMDCs is differentially regulated by various hCG preparations. Another murine study has recently analyzed the effect of hCG at the fetal–maternal interface during late gestation. The authors could not detect alterations in IFN-γ, IL-2, IL-4, IL-5, IL-6, IL-10, IL-12p70, and TNF-α plasma concentrations after *in vivo* application of hCG. However, hCG administration provoked an increase in the proportion of decidual Treg cells (30). Two human studies generated DCs from blood-derived monocytes and reported controversial outcomes. While Segerer and colleagues proved an effect of hCG on HLA-DR upregulation which was inhibited in stimulated monocytes-derived DCs as well as a reduced T cell stimulatory capacity (18), Huck and colleagues found no significant changes in surface marker expression, cytokine secretion, or T cell stimulatory capacity in the presence of β-hCG (15). Moreover, Yoshimura and colleagues investigated the influence of hCG on myeloid and lymphoid DC subsets obtained from human peripheral blood. The authors observed an increment in the expression of maturation markers, costimulatory molecules, and inflammatory cytokines, as well as an elevated capacity to stimulate allogeneic immune responses (21). Unfortunately, most studies did not clearly indicate the origin of the hCG preparation they used impeding any conclusions about the different outcomes provoked by the different hCG preparations. Additionally, hCG-mediated effects may differ between the species. Thus, follow-up studies are needed to finally clarify the effects of rhCG and uhCG on human cells as well as underlying signaling pathways. Molecules, such as IL-6, IFN-γ, and indoleamine 2,3-dioxygenase, were suggested to be involved in hCG-initiated signaling in DCs and might be involved in the induction of tolerogenic DCs (19, 20).

Next, we assessed whether hCG may influence T cell differentiation from naïve T cells indirectly by modulating BMDCs. Our results revealed that treatment of BMDCs with rhCG prior to cocultures with stimulated naïve T cells did neither affect TH1, TH2, nor TH17 cell differentiation. Wan and colleagues reported no influence of uhCG on the TH1 and TH2 polarization capacity of BMDCs on naïve T cells by determining IFN-γ and IL-4 production. However, the authors detected a significant elevated IL-10 production in cocultures between naïve T cells and hCG-treated BMDCs, when compared to cocultures without hCG treatment (20). This could not be observed in our experiments suggesting that uhCG and rhCG not only differ in respect to their influence on the cytokine profile of BMDCs but also on the T cell polarization capacity. These observations might be of interest for the usage of hCG preparations in patients undergoing assisted reproductive techniques. uhCG and rhCG preparations are usually applied for ovulation induction and luteal support. Depending on the applied hCG preparation, the immune system of the patient may react differentially. Several studies compared the efficacy of uhCG and rhCG in assisted reproductive techniques. Recently, Youssef and colleagues performed a meta-analysis to assess the potency of both hCG preparations in subfertile women undergoing *in vitro* fertilization and intra cytoplasmic sperm injection cycles. The authors found no evidence for a difference between rhCG and uhCG concerning live birth or ongoing pregnancy rates (31).

It has been proposed that hCG treatment might be helpful in the prevention of threatened abortions (32). Here, we addressed the question whether the adoptive transfer of hCG-modulated BMDCs may prevent fetal rejection in a murine abortion-prone model. In a first set of experiments, hCG-treated or mature BMDCs were adoptively transferred at very early pregnancy stages according to our previously used protocol for adoptive transfer of Treg cells (23). Adoptive transfer of hCG-treated BMDCs elevated Treg cell numbers directly at the fetal–maternal interface. However, this did not result in changes of the abortion rate or number of implantations. Thus, we assumed that adoptive transfer of BMDCs was too late to influence pregnancy outcome. Next, we adoptively transferred BMDCs to abortion-prone females prior to mating. Only those females that had a plug within the next 3 days following BMDC treatment were included in the study. Transfer of hCG-treated BMDCs could diminish the abortion rate compared to females that had been transferred with mature BMDCs. This effect was accompanied by a significant augmentation of the peripheral and uterine Treg number in the hCG-treated BMDC group that may account for the observed effect on fetal survival. Using a similar mouse model, Blois and colleagues adoptively transferred syngeneic BMDCs into abortion-prone females and showed a significant reduction in fetal resorption, which was associated with decreased serum IL-6 levels (33). Unfortunately, the authors did not test whether the Treg cell number was changed after BMDC transfer. However, in a follow-up study, Miranda and colleagues showed that syngeneic DC therapy increased the number of CD8 and γδ cells and induced TGF-β1 and PIBF expression (34). In line, transfer of hCG-treated BMDCs provoked an elevation of IL-10 and TGF-β in decidual tissue. Therefore, it is tempting to speculate that transfer of tolerogenic BMDCs induced a local elevation of T cell populations with regulatory properties producing pregnancy-protective cytokines like IL-10 and TGF-β (35). An early Treg cell augmentation is a prerequisite for proper blastocyst implantation, as depletion of Treg cells prior to mating has been associated with implantation failure (25). Treg cell deficiency resulted in uterine inflammation and fibrosis suggesting that at this early pregnancy time point Treg cells may counteract pro-inflammatory events occurring during implantation and thereby create a uterine environment that supports nidation of the embryo. As anti-inflammatory cytokines, we propose IL-10 and TGF-β to be involved in this process.

Interestingly, Blois and colleagues found that fetal protection was short lived as females that became pregnant more than 2 days after transfer had abortions while females that became pregnant during the first 2 days had no abortions. In our present study, we could not observe a time-dependent effect between BMDC transfer and pregnancy establishment. However, we found that BMDCs that were transferred after mating did not affect fetal resorption suggesting that additionally to a short-lived effect, protective mechanisms induced by DCs have to take place very early in pregnancy.

In conclusion, our results propose a direct effect of rhCG on DCs by retaining these cells in a rather immature, thus tolerogenic state *in vitro*. Moreover, we showed for the first time that *in vitro* rhCG-modulated DCs are fully functional *in vivo*. When adoptively transferred prior to mating, hCG-modulated DCs increased Treg cell numbers and protected fetuses from rejection. Thus, our data contribute to a better understanding on the *in vitro* and *in vivo* effects of hCG on DCs. In normal fertile women, hCG levels rapidly increase after blastocyst implantation. Before pregnancy, in the pre-ovulatory phase of the menstrual cycle, small quantities of pituitary hCG can be detected. Pituitary hCG has been reported to possess physical, immunological, and biological similarities to placental hCG (36). However, until today, there is no evidence that pituitary hCG can modulate DCs even before pregnancy arises and support successful pregnancy establishment. Thus, further studies are required to validate our observations made in the murine system for normal fertile women as humans and mice display some species specific differences in reproductive processes (37). However, our findings support the usage of hCG in the treatment of subfertile patients where hCG is applied before fertilization to induce final oocyte maturation. Additionally, hCG preparations obtained from different sources seem to affect DCs differentially. Therefore, the clinical application of hCG should be taken with caution.

## Author Contributions

DD, SE, and SL performed and analyzed experiments. AS and ACZ designed and supervised experiments. DD and AS prepared figures, interpreted data, and wrote the manuscript. ACZ, SL, and SE critically revised the manuscript.

## Conflict of Interest Statement

The authors declare that the research was conducted in the absence of any commercial or financial relationships that could be construed as a potential conflict of interest.
